# Development of a biocompatible 3D hydrogel scaffold using continuous liquid interface production for the delivery of cell therapies to treat recurrent glioblastoma

**DOI:** 10.1002/btm2.10676

**Published:** 2024-07-30

**Authors:** Lauren Kass, Morrent Thang, Yu Zhang, Cathleen DeVane, Julia Logan, Addis Tessema, Jillian Perry, Shawn Hingtgen

**Affiliations:** ^1^ Division of Pharmacoengineering and Molecular Pharmaceutics, UNC Eshelman School of Pharmacy The University of North Carolina at Chapel Hill Chapel Hill North Carolina USA; ^2^ Department of Chemistry, UNC College of Arts and Sciences The University of North Carolina at Chapel Hill Chapel Hill North Carolina USA; ^3^ Center for Nanotechnology in Drug Delivery, Eshelman School of Pharmacy The University of North Carolina at Chapel Hill Chapel Hill North Carolina USA; ^4^ Lineberger Comprehensive Cancer Center The University of North Carolina at Chapel Hill Chapel Hill North Carolina USA

**Keywords:** 3D printing, cell therapy, CLIP, glioblastoma, scaffold

## Abstract

Glioblastoma (GBM) is the most common primary malignant brain tumor diagnosed in adults, carrying with it an extremely poor prognosis and limited options for effective treatment. Various cell therapies have emerged as promising candidates for GBM treatment but fail in the clinic due to poor tumor trafficking, poor transplantation efficiency, and high systemic toxicity. In this study, we design, characterize, and test a 3D‐printed cell delivery platform that can enhance the survival of therapeutic cells implanted in the GBM resection cavity. Using continuous liquid interface production (CLIP) to generate a biocompatible 3D hydrogel, we demonstrate that we can effectively seed neural stem cells (NSCs) onto the surface of the hydrogel, and that the cells can proliferate to high densities when cultured for 14 days *in vitro*. We show that NSCs seeded on CLIP scaffolds persist longer than freely injected cells in vivo, proliferating to 20% higher than their original density in 6 days after implantation. Finally, we demonstrate that therapeutic fibroblasts seeded on CLIP more effectively suppress tumor growth and extend survival in a mouse model of LN229 GBM resection compared to the scaffold or therapeutic cells alone. These promising results demonstrate the potential to leverage CLIP to design hydrogels with various features to control the delivery of different types of cell therapies. Future work will include a more thorough evaluation of the immunological response to the material and improvement of the printing resolution for biocompatible aqueous resins.

**Translational Impact Statement**

Glioblastoma is a deadly brain tumor with dismal survival outcomes. Patients are in desperate need of new strategies to treat glioblastoma, particularly to prevent recurrence after surgical resection. Cell therapies are gaining traction in the clinic as more effective cancer‐killing agents than traditional chemotherapies. However, translation of cell therapies to the clinic hinges on optimized delivery to minimize toxicity and maximize activity. Here, we develop a novel delivery system to enable enhanced transplantation and survival time of therapeutic cells for the treatment of recurrent glioblastoma.

## 1 INTRODUCTION

Glioblastoma (GBM) is the deadliest malignant primary brain tumor occurring in adults.^1^ Standard of care treatment for GBM, resection surgery followed by concomitant radiation and temozolomide chemotherapy, has not evolved in nearly 20 years.^2^, ^3^ The prognosis for patients even with standard of care treatment strategies remains extremely poor, at a median survival of merely 12–15 months.^4^ GBM tumors have a proclivity to invade areas of the brain adjacent to the primary tumor, posing the threat of tumor recurrence even after resection surgery, with most recurrent tumors growing within 2 cm of the primary tumor mass.^5^ Even with post‐surgical treatments intended to mitigate local and distant tumor recurrence,^6^ these still occur for 90% of GBM patients.^7^ Thus, it is clear that the development of more effective post‐surgical GBM therapies is vital for reducing recurrence rates and improving patient survival.

Cell therapies are promising candidates for improving outcomes for GBM patients. Many cells have been explored pre‐clinically and clinically for cancer treatment. Neural stem cells (NSCs),^8^, ^9^ mesenchymal stem cells (MSCs),^10^ and fibroblasts,^11^ among other cell types, have been genetically engineered to constitutively secrete anti‐cancer drugs. Additionally, CAR‐T cells (CAR‐T),^12^ CAR‐macrophages (CAR‐M),^13^ or cell‐based anti‐cancer vaccines^14^ can be employed as immunotherapy approaches to treat cancer. Cell therapies have high potential for success in the clinic, with marked improvements over traditional small molecule or biologic treatment strategies. Whole cells offer greater site selectivity and specificity, enhanced transport and distribution in the body with little impact from genetic variability, and the potential for higher overall potency and lower off‐target toxicity. However, novel cell therapies often fail in clinical trials due in part to issues that could be ameliorated by enhanced delivery approaches. Some cell therapies, like NSC‐ and MSC‐based therapies, suffer from low persistence at the site of implantation, limiting the therapeutic durability of the cells.^15^, ^16^ Others, like CAR‐T, CAR‐M, and whole‐cell vaccine therapies can have difficulty trafficking to and infiltrating the tumor site, and can also induce systemic toxicity and off‐target effects if not delivered locally.^12^, ^13^, ^17^ Thus, a locally implanted, biomaterial‐based cell delivery platform could solve some of these issues facing clinical implementation of various cell therapies.

While many cell delivery biomaterial systems exist in literature, few have been optimized for the delivery of anti‐cancer cell therapies. Many published studies utilize cell‐biomaterial platforms for tissue engineering and regenerative medicine, in which the overarching goal is to keep the cells in a localized area and guide their growth into healthy, functional tissue.^18^ However, cell therapies indicated for cancer usually incorporate a trafficking or drug release component, in which the cells or secreted macromolecules must exit from a local delivery system to reach the nearby tumor. Thus, existing systems used for cell transplantation in tissue engineering applications are unlikely to improve the delivery of cell therapies for cancer treatment.

Injectable hydrogels are a popular option for the delivery of cell therapies for various applications. However, injectable systems have several notable disadvantages compared to 3D printed implantable systems, specifically for the treatment of GBM. First, injectable hydrogels must be prepared individually at the time of treatment. This means that cells usually cannot be cultured in the biomaterial system prior to *in vivo* administration, despite pre‐culture time having a positive effect on *in vivo* persistence of the encapsulated cells.^19^ By contrast, a 3D printed system that can be easily handled between production and surgical implantation allows for the priming of the therapeutic cells in their new niche *in vitro* prior to delivery *in vivo*. Second, because 3D printed hydrogels can be prepared and seeded with cells in advance of the time of surgery, this simplifies the implantation procedure. In contrast, the gelation kinetics of injectable systems must be carefully optimized. If gelation occurs too early, solidified hydrogel can cause clogging in the syringe; if gelation occurs too late, cells can leak prematurely into the area surrounding the implant site, lacking the protection afforded by the material. Finally, while injectable systems are preferred for non‐invasive delivery, the administration of the cells into the GBM resection cavity at the time of resection surgery would be ideal so that the cells are delivered directly to the tissue sites most likely to contain residual tumor lesions. Thus, the benefit of non‐invasive delivery for this application is insignificant.

**FIGURE 1:**
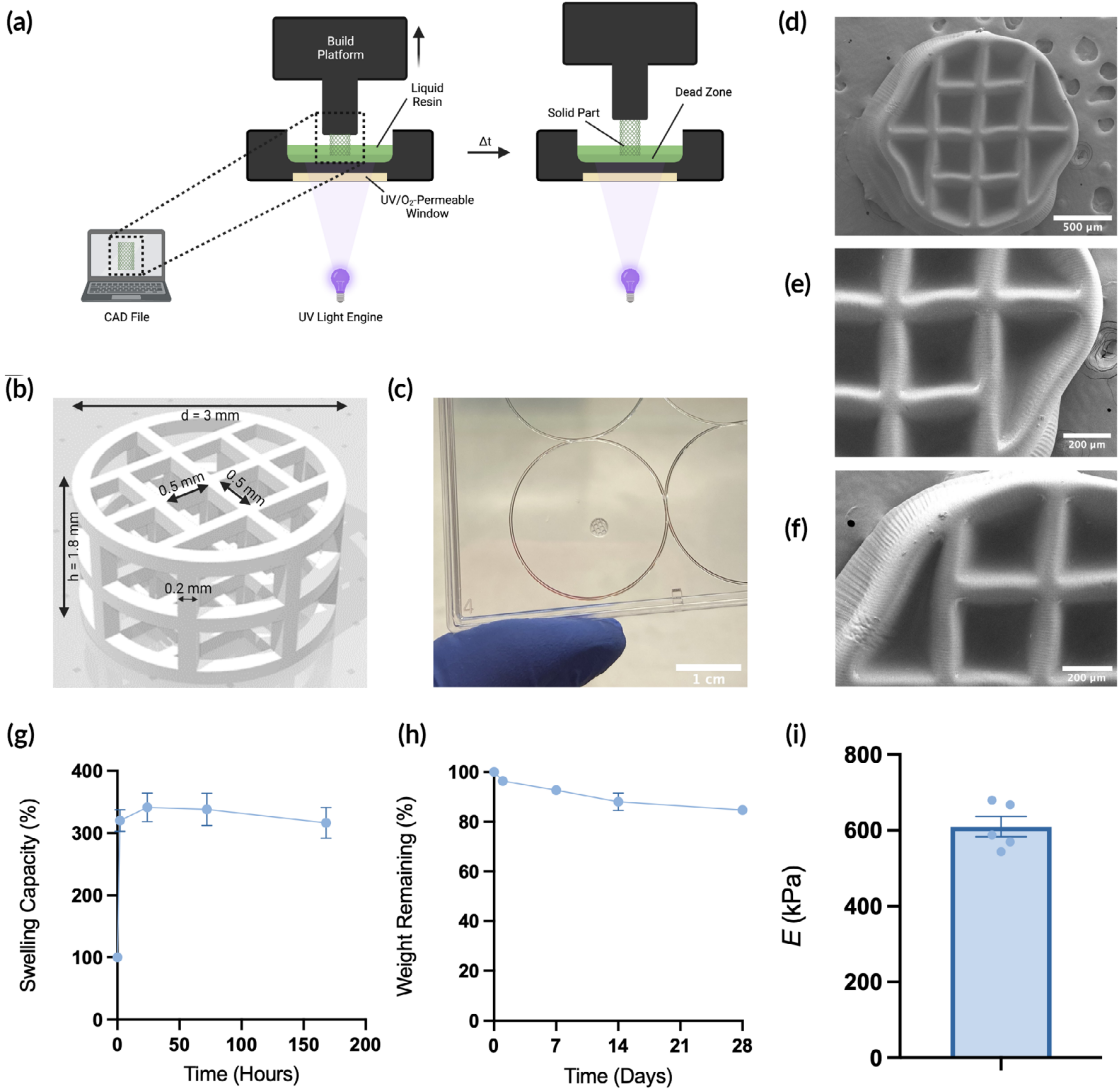
Fabrication and characterization of 3D hydrogels made with continuous liquid interface production (CLIP). (a) Schematic of CLIP process. (b) 3D rendering of the lattice scaffold design. (c) Photograph of CLIP scaffold. (d–f) SEM micrographs of acellular CLIP scaffolds. (g) Swelling capacity of bulk hydrogels (*n* = 5). (h) Degradation profile of bulk hydrogels (*n* = 3). (i) Young's modulus of printed resin samples (*n* = 5).

We hypothesized that we could develop a 3D‐printed hydrogel system that could be utilized for cell delivery using continuous liquid interface production (CLIP). CLIP is a monolithic 3D printing strategy based on digital light synthesis, in which a liquid photoreactive resin is polymerized into a solid with UV light (Figure 1a).^20^ Polymerization occurs at precise locations to build a custom 3D structure from a computer‐aided design (CAD) file, allowing facile customization of the 3D architecture. The part is built in an inverted manner with the base attached to the build platform, which lifts the solid part out of the resin reservoir as polymerization continues. Unique to CLIP is that the window through which UV light polymerizes the liquid resin is also permeable to oxygen, which acts as a free radical scavenger in a thin layer above the window referred to as the “dead zone.” The dead zone acts as a continuous source of liquid resin from which the part is polymerized, allowing the printing process to proceed continuously and without intermediate processing steps required of traditional stereolithography (SLA) printers. This greatly improves the overall efficiency of the 3D printing process, allowing for rapid optimization of various resin formulations and 3D designs.

We postulated that by building a protective and biocompatible 3D scaffold onto which therapeutic cells can be seeded, transplantation efficiency would be increased without preventing cell migration to nearby tumors, thus improving efficacy and safety. However, the use of CLIP to develop such a delivery system has not yet been performed. As such, characterization of cell viability and function when seeded on scaffolds manufactured using CLIP is necessary.

In this study, we test our hypothesis using two cell therapies in the context of recurrent glioblastoma. We first develop a biocompatible resin for use in printing a 3D scaffold with CLIP. Next, we test the compatibility of our novel hydrogel delivery system *in vitro* with HB1.F3 NSCs, which have been extensively characterized in literature for their unique tumor‐homing properties^8^, ^21^, ^22^, ^23^—which can be leveraged for enhanced drug delivery—and are currently in clinical trials for potential use in brain cancer treatment (NCT02015819, NCT02055196, NCT02192359, NCT01172964, NCT03072134).^24^, ^25^, ^26^, ^27^, ^28^ We show that the cells can be seeded and grown on our novel scaffolds, and that their migratory ability—a key component of their therapeutic activity—is not hindered by the scaffold *in vitro*. We then investigate the ability for these cells to persist *in vivo* using our model of mock GBM resection in mice. Finally, we test our novel system in a recurrent GBM mouse model using normal human fibroblasts (NHF1s) engineered to secrete the anti‐tumor protein, TNF related apoptosis‐inducing ligand (TRAIL), seeded onto the novel scaffolds. These cells, though non‐tumor‐homing,^29^ act as a constitutive drug pump of TRAIL, which we hypothesized would easily diffuse to tumor lesions from the cells attached to and protected by CLIP scaffolds.

## 2 METHODS

### 2.1 Materials and cell lines

Poly(ethylene glycol) diacrylate (PEGDA, *M*_n_ = 700) was purchased from Sigma Aldrich, gelatin methacryloyl (GelMA, bloom strength = 300, degree of methacrylation = 45%–55%) was purchased from Cellink, and UV radical initiator lithium phenyl‐2,4,6‐trimethylbenzoylphosphinate (LAP) was purchased from TCI Chemicals. Sterile water was purchased from Corning.

HB1.F3.CD cells were obtained from Dr. Karen Aboody (City of Hope National Medical Center). Briefly, HB1.F3 NSCs from primary fetal telencephalon cultures at 15 weeks of gestation were v‐myc immortalized, transduced with a retroviral vector pMSCV‐puro/CD, and expanded.^30^, ^31^ HB1.F3.CD subclone 21 was given to the University of North Carolina under a material transfer agreement and cultured as previously described.^32^, ^33^ LN229 cells were obtained from the American Type Culture Collection. NHF1 cells were obtained from W. Kauffman (University of North Carolina School of Medicine) and were hTERT‐immortalized. All aforementioned cells were cultured using Dulbecco's Modified Eagle Medium (Gibco) with 10% fetal bovine serum (FBS) and 1% penicillin/streptomycin, hereby referred to as standard culture media.

### 2.2 Lentiviral transduction

Cells were transduced to facilitate in vitro and in vivo tracking via luminescence or fluorescence reporters, and/or to facilitate expression of therapeutic proteins. Transduction for all cell lines was performed by incubating cells with 8 μg/mL polybrene and the lentiviruses for 24 h at 37°C and 5% CO_2_. HB1.F3.CD cells were transduced with lentiviruses encoding for green fluorescent protein (GFP) and firefly luciferase (Fluc), hereby referred to as HB1^Fluc^. NHF1 fibroblasts were transduced with lentiviruses encoding TRAIL, hereby referred to as NHF1^TRAIL^. LN229 cells were transduced with lentiviruses encoding mCherry (mCh) and Fluc, hereby referred to as LN229^Fluc^. All lentiviruses were purchased from the Duke Viral Vector Core.

### 2.3 Fabrication of CLIP scaffolds

CLIP scaffolds were produced using the S1 CLIP prototype printer (Carbon) utilizing a 385 nm LED UV light source. The scaffold design was built in SolidWorks® and resulting STL files were sliced at 5 μm using the Carbon printing software. The scaffold dimensions are detailed in Figure 1b.

The resin was prepared by stirring 14 wt% PEGDA, 10 wt% GelMA, 0.25 wt% LAP, and 75.75 wt% standard culture media at 60°C until fully dissolved. Scaffolds were printed in a 4 × 8 array at a continuous speed of 24 mm/h and light intensity of 1 mW/cm^2^. After each print, excess resin was gently removed from the scaffolds using compressed air and deionized (DI) water. Scaffolds were stored in fresh DI water in a biosafety cabinet and sterilized by UV irradiation for 30 min prior to use.

### 2.4 Characterization of CLIP scaffold material properties

#### 2.4.1 Degradation rate

Bulk hydrogels were prepared by curing resin in rectangular molds for 5 min using a UV lamp (365 nm, AnalytikJena UVGL‐58). Hydrogels were then washed for 24 h at room temperature in DI water on a rotating mixer. Samples were dried in an oven at 37°C for 3 days, then weighed to obtain the initial dry weight (*W*_i_). Samples were then immersed in 5 mL tubes containing 1 mL of 0.5 mg/mL type 1 collagenase (Sigma Aldrich) in 1× PBS (Gibco) and incubated at 37°C and 5% CO_2_ for 28 days (*n* = 3 per time point). Collagenase solution was replaced twice per week to maintain enzyme activity. At specified time points between 0 and 28 days, collagenase solution was replaced with 2 mL of DI water and samples were washed on a rotating mixer at room temperature for 48 h. Samples were then dried in an oven at 37°C for 3 days and weighed (*W*_d_) to obtain the percent weight loss:

(1) $$\text{Percent degradation}=\frac{W_{i}-W_{d}}{W_{i}}\times 100\%$$

#### 2.4.2 Swelling rate

Bulk hydrogels were prepared, washed, and dried as described in the preceding section (*n* = 3). Samples were weighed to obtain the initial dry weight, then immersed in a 6‐well plate containing 3 mL DI water per well and incubated at 37°C and 5% CO_2_ for 7 days. At specified time points between 0 and 7 days, swollen hydrogels were removed from the tubes and weighed to obtain the percent swelling:

(2) $$\text{Percent Swelling}=\frac{W_{s}-W_{i}}{W_{i}}\times 100\%$$

Hydrogels were weighed at each time point, then returned to the tubes with fresh DI water at 37°C and 5% CO_2_ until the next time point.

#### 2.4.3 Toxicity of leachable and degradation products

Scaffolds were prepared and sterilized as described above, then added to 2 mL centrifuge tubes (*n* = 3 per time point) containing 200 μL 1X PBS or 1X PBS with 0.5 mg/mL type 1 collagenase and incubated at 37°C and 5% CO_2_ for 112 days. For samples incubated with collagenase, the media was changed twice per week to maintain enzyme activity. At specified time points between 0 and 112 days, scaffolds were removed from the tubes and the media was stored at 4°C until testing. To test for toxicity, HB1^Fluc^ cells were seeded into a 96‐well plate at a density of 5 × 10^3^ cells per well. After a 24‐h equilibration period, the medias from the scaffold incubations were used to treat the cells at a ratio of 1:3 with standard culture media. Fresh 1X PBS and 1X PBS containing 0.5 mg/mL type 1 collagenase served as controls. The cells were incubated with the samples for 24 h, after which the media was replaced with 0.75 mg/mL D‐luciferin (PerkinElmer 122799) in 1X PBS, which reacts only with viable HB1^Fluc^ cells. Bioluminescence (BLI) signal was quantified using the *in vivo* imaging system (IVIS) Spectrum.

#### 2.4.4 Young's modulus

Thin sheets 1 mm thick were printed using the same resin and printing conditions as described above, then swollen to equilibrium in deionized water. Dogbone‐shaped samples were cut from the thin sheets using a mold with bridge dimensions 12 mm × 2 mm. Samples were loaded into an RSA‐G2 dynamic mechanical analysis (DMA) system (TA Instruments) and stretched uniaxially at a constant strain rate of 0.005 s^−1^ until breaking point.

### 2.5 Seeding cells onto CLIP scaffolds

#### 2.5.1 Seeding method

Sterile scaffolds were removed from storage in DI water and placed individually into sterile 2 mL centrifuge tubes. Excess liquid was pipetted off the surface of the scaffold prior to seeding. A total of 1 × 10^6^ HB1^Fluc^ or NHF1^TRAIL^ in 3 μL of 1X PBS was gently pipetted onto the top surface of each scaffold. The scaffolds were then incubated at 37°C and 5% CO_2_ for 45 min, then centrifuged at 1500 RPM for 6 min. Scaffolds were then stored in standard culture media at 37°C and 5% CO_2_ for long‐term culture.

#### 2.5.2 Seeding efficiency

Seeding efficiency was determined using a genomic DNA extraction kit (ThermoFisher K182002). A standard curve of HB1^Fluc^ cell number versus DNA concentration was obtained by resuspending HB1^Fluc^ cells at various counts in the range of 5 × 10^4^–2 × 10^6^ cells per tube (*n* = 3). Genomic DNA was then extracted from each tube per manufacturer's instructions, and the DNA concentration was quantified using the Qubit Fluorometric Quantification system (ThermoFisher). To quantify scaffold seeding efficiency, genomic DNA was extracted from scaffolds, seeded as described above, using the same method (*n* = 3). The standard curve was used to calculate the corresponding cell number for each scaffold, and seeding efficiency was quantified:

(3) $$\text{Seeding Efficiency}=\frac{\text{Observed Cell Count}}{1\times 10^{6}}\times 100\%$$

### 2.6 Scanning electron microscopy

Scaffolds (*n* = 3 per time point) were seeded as described above, then fixed in 10% formalin overnight. Fixed scaffolds were then dehydrated by serial ethanol dilution in 50%, 75%, 90%, and 100% ethanol in water for 5 min each, then dried using a critical point drier (Tousimis Autosamdi‐931). Scaffolds were affixed to aluminum stubs using double‐sided carbon tape and coated with 11 nm of gold–palladium using a sputter coater (Cressington 108auto). Images were obtained using the Hitachi S‐4700 SEM with 2 kV accelerating voltage.

### 2.7 *In vitro* characterization of cells on CLIP scaffolds

#### 2.7.1 Viability

Scaffolds were seeded as described above with HB1^Fluc^ and placed individually into 6‐well plates with 5 mL of standard culture media per well. The scaffolds were cultured at 37°C and 5% CO_2_ for 14 days, with media changes occurring every other day. On days 0, 1, 3, 5, 7, and 14, scaffolds (*n* = 3 per time point) were removed from the culture plates and placed in a new 6‐well plate. The scaffolds were submerged in 15 mg/mL D‐luciferin in PBS and BLI signal was quantified using the IVIS spectrum.

#### 2.7.2 Migration

Scaffolds (*n* = 3) were seeded as described above with 1 × 10^6^ HB1^Fluc^ and cultured at 37°C and 5% CO_2_ in standard culture media for 5 days. On day 4, a 0.6% agarose solution was prepared in standard culture media. The solution was microwaved to dissolve the agarose, and 2 mL of the solution was pipetted into each well of a 6‐well plate. PLA microfibers mimicking the white matter migratory paths in the brain were dispersed randomly in each well before the agarose solidified. The solutions were allowed to cool and solidify, then 3 uL containing 1 × 10^6^ LN229^Fluc^ cells were injected into the resulting gel using a Hamilton syringe. 1 mL of standard culture media was added to the top of each gel, and the LN229^Fluc^ cells were cultured in the plates at 37°C and 5% CO_2_ overnight. The next day, a small portion of the agarose gel adjacent to the LN229^Fluc^ cells was vacuum aspirated, and the HB1^Fluc^‐laden scaffolds were implanted into the resulting cavity. 150 uL of 0.6% agarose in standard culture media was warmed to 45°C and gently pipetted on top of the scaffold and allowed to cool to seal the scaffold in place. 1 mL of standard culture media was added to the top of the gel, and the plates were cultured for 1 week at 37°C and 5% CO_2_ with media changes performed every other day. Wells in which seeded CLIP scaffolds were implanted without the presence of tumor served as a negative control. Fluorescence microscopy was used to evaluate HB1^Fluc^ migration over time. Images of green and red fluorescence channels were merged using ImageJ.

### 2.8 *In vivo* scaffold biocompatibility and HB1^Fluc^ persistence

Animal studies were approved by the University of North Carolina at Chapel Hill's Animal Care and Use Committee. Female, athymic nude mice (Animal Studies Core, University of North Carolina at Chapel Hill) aged 6–8 weeks were used for all studies. The animals were first anesthetized using 2.5% inhaled isoflurane, then placed into a stereotactic frame. The surgical site was prepared using antiseptics betadine and 70% isopropyl alcohol. The skull was then exposed with a small incision made in the skin on the head of the mouse. A craniotomy was then performed using a microdrill to remove a small portion of the parietal skull plate, 3 mm in diameter, between the bregma and lambda points in the right hemisphere of the brain. Bleeding was controlled using cold saline, and the wound was closed using Vetbond (3M, 1469SB) after bleeding had subsided. Post‐operative pain management was performed using subcutaneous injection of 5 mg/kg meloxicam, 24‐ and 48‐h post‐surgery. 1 week after the craniotomy, implantation of cells and/or scaffolds was performed. The mice were prepared for surgery as described above, and the surgical site was reopened. The dura mater was removed using a 25 G needle, and a vacuum pump was used to make a mock resection cavity approximately 3 mm in diameter and 2 mm in height. Bleeding was controlled with cold saline and GelFoam® (Pfizer), when needed. After bleeding had subsided, acellular CLIP scaffolds (for biocompatibility studies, *n* = 3) or 1 × 10^6^ HB1^Fluc^ cells suspended in 3 μL 1X PBS or seeded on CLIP scaffolds (for persistence studies, *n* = 4–5) were implanted into the cavity. The wound was closed with Vetbond and pain was managed via 5 mg/kg subcutaneous meloxicam 24‐ and 48‐h post‐surgery. For biocompatibility studies, the mice were monitored after scaffold implantation for 1 month, after which histology was performed. For persistence studies, serial BLI imaging was performed using the IVIS spectrum with 150 mg/kg intraperitoneal injection of D‐luciferin in 1X PBS.

### 2.9 Histology

Mice were anesthetized with 5% inhaled isoflurane before cardiac perfusion was performed by injection of 5 mL of 1× PBS then 5 mL 10% formalin into the left ventricle. After cervical dislocation, the brain was dissected and immediately stored in 10% formalin. After 48 h of fixation in formalin, trimmed tissues were embedded in paraffin, sliced into 5 μm sections, and stained with hematoxylin and eosin (H&E) using an Autostainer XL from Leica Biosystems. Stained slides were imaged at 20X with an Aperio ScanScope XT from Leica Biosystems.

### 2.10 *In vivo* NHF1^TRAIL^ efficacy

Craniotomies were performed as described above. 1 week after the craniotomy, mice (*n* = 7–8) were prepared for tumor cell injections. The wound was reopened and 1 × 10^5^ LN229^Fluc^ cells suspended in 3 μL 1X PBS containing 10% Matrigel were injected into the brain parenchyma using a stereotactic auto‐injector. The injections were performed at a rate of 1 μL/min at stereotactic coordinates 2.5, −0.5, −0.5 from the bregma, and avoiding the lateral ventricles. The syringe was slowly removed 5 min after the injection was complete to avoid reflux of the cell suspension. The wound was then closed with Vetbond and post‐operative pain was managed via 5 mg/kg subcutaneous meloxicam injection, 24‐ and 48‐h after surgery. 7 days after tumor implants, the mice were prepared for tumor resection and stem cell implantation. Prior to surgery, mice were randomized into groups exhibiting statistically similar mean total flux values based on tumor BLI imaging performed immediately preceding resection. The wound was reopened, and tumors were resected using a vacuum pump and fluorescence imaging as guidance. Next, after bleeding had subsided, 5 × 10^5^ NHF1^TRAIL^ cells suspended in 3 μL 1X PBS or seeded on CLIP scaffolds were implanted into the resection cavity. Acellular CLIP scaffolds served as a negative control. The wounds were closed using Vetbond, and post‐operative pain was managed via 5 mg/kg subcutaneous meloxicam injection, 24‐ and 48‐h after surgery. BLI imaging with the IVIS Spectrum and 150 mg/kg D‐luciferin in 1X PBS via intraperitoneal injection was used to track tumor volume. Mice were euthanized when more than 20% of their original body weight was lost or if other pain‐related symptoms, such as dehydration, hunched position, tremors, and low body temperature, were observed.

### 2.11 Statistical analysis

All results are presented as mean ± standard error of the mean. In vitro toxicity data was analyzed with one‐way ANOVA and Dunnett's multiple comparison's correction. Persistence data was analyzed with Student's *t* test. For all analyses, ns indicates not significant, * indicates *p* < 0.05, ** indicates *p* < 0.01, *** indicates *p* < 0.001, and **** indicates *p* < 0.0001. Statistical analyses were conducted using Prism GraphPad (version 9).

## 3 RESULTS

### 3.1 Fabrication and characterization of 3D composite CLIP hydrogels

An ideal biomaterial delivery system for anti‐cancer cell therapy would be capable of simultaneously protecting and controlling the release of cells to ensure a sufficient dose of live cells over a sustained period. Previously tested systems for use in GBM therapy demonstrated that NSCs physically encapsulated within the material could not provide a significant improvement in therapeutic efficacy compared to controls.^15^ This phenomenon may be attributed to a reduction in cell migration from the material implant into the surrounding diseased tissue due to inherent material properties, such as slow degradation or small pore size. Contrastingly, we hypothesize that by seeding cells onto the external surfaces of a 3D structure, cells or macromolecules may be released from the implant readily while the hydrogel acts as a physical barrier to cell clearance. Thus, we considered the design criteria of a 3D hydrogel, fabricated with CLIP, for this purpose. The design criteria were (1) printability, (2) biocompatibility, and (3) compatibility with the maximum resection cavity shape and size that can be made safely in our mouse model of GBM resection and recurrence.

To satisfy condition (1), we prepared the hydrogel resin with 14 wt% PEGDA, 10% GelMA, and 0.25 wt% LAP in water. Compatibility with the CLIP process (Figure 1a) requires that the resin is liquid at room temperature, photoreactive, and is not significantly over‐ or under‐cured—that is, structural integrity and the architectural features of the designed shape are maintained in the resulting part. Condition (2) was satisfied by the incorporation of 10% GelMA in the resin, which serves as a cell‐adhesive polymer containing RGD adhesion sites onto which the cells can attach. Moreover, the resin does not contain highly toxic co‐polymers, initiators, or solvents, and only limited cytotoxicity from leached acrylates and LAP was expected. The 3D structure shown in Figure 1b satisfies condition (3). The cylindrical lattice design fits within the maximum resection cavity dimensions (diameter = 3 mm and height = 2 mm). Figure 1c is a photograph of the CLIP‐printed hydrogel with the previously described resin. Upon closer inspection of the hydrogel features shown by SEM images in Figure 1d–f, it was observed that the general features of the scaffold in the XY‐plane are conserved in the printing process (apart from shrinkage due to sample dehydration prior to imaging). However, overcuring of the part is visible—the void spaces of the lattice do not penetrate through the other face of the scaffold—resulting in pockets rather than pores. Though this artifact of the printing process was observed, this feature proved advantageous for cell seeding and culture, as discussed below.

Degradation of the bulk scaffold material was minimal, with only 15% weight loss observed after 4 weeks in PBS containing collagenase (Figure 1h). Completely dry bulk hydrogel samples swell to nearly 350% of their initial weight and reach equilibrium swelling after 24 h (Figure 1g), though the swelling rate would expectedly increase for a sample with higher surface area. Despite the high swelling capacity of completely dry samples, the equilibrium swelling of hydrogel samples after curing is minimal, swelling only 2.43 ± 1.15% higher than its initial printed weight. The Young's modulus of the material, as measured by DMA, was 609.9 ± 27.1 kPa (Figure 1i).

### 3.2 *In vitro* and *in vivo* biocompatibility of CLIP scaffolds

**FIGURE 2:**
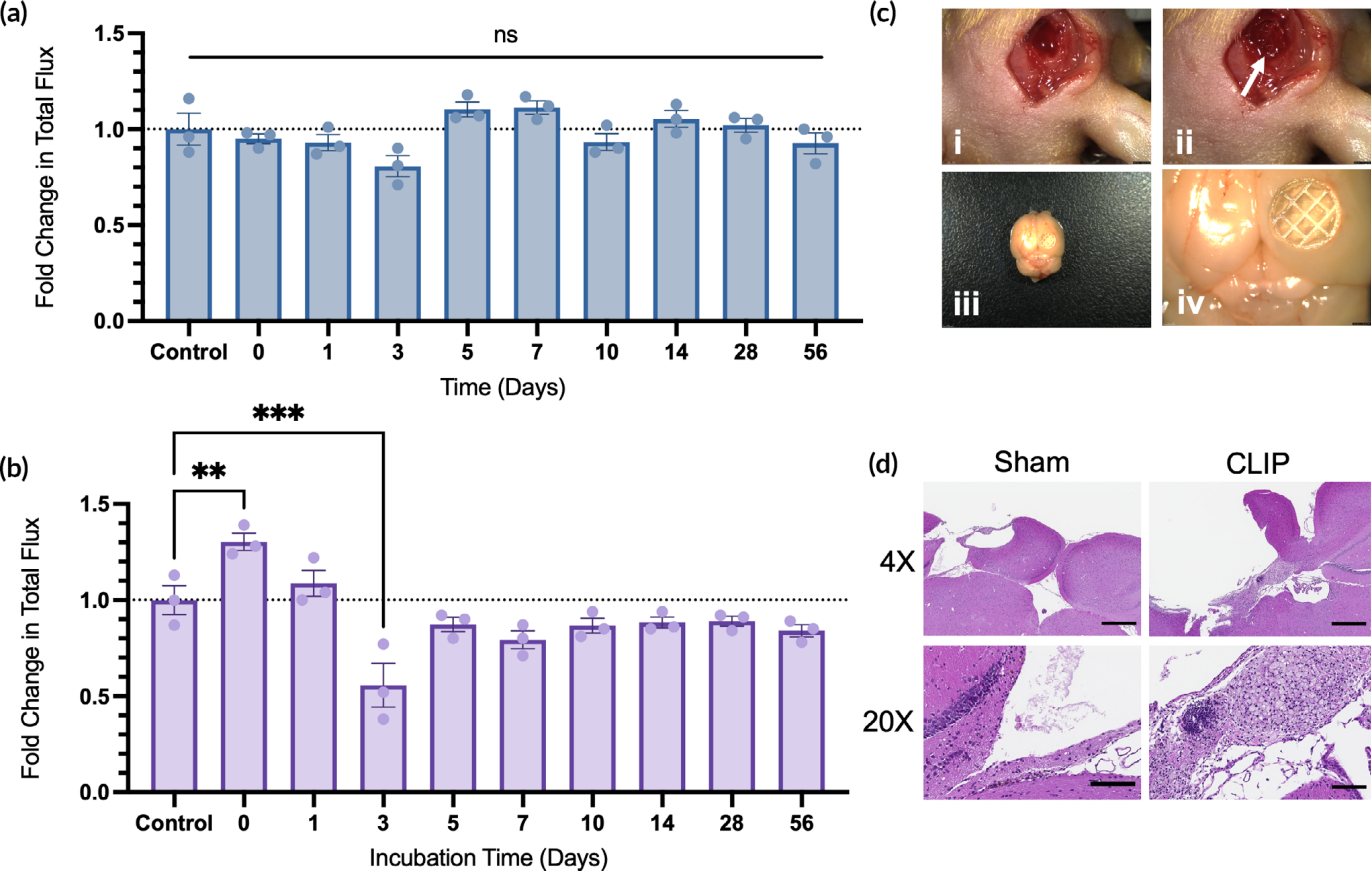
Characterization of *in vitro* and *in vivo* biocompatibility. (a) Toxicity of leachable products from acellular continuous liquid interface production (CLIP) scaffolds on HB1^Fluc^ cells incubated in PBS (*n* = 3). (b) Toxicity of degradation products from acellular CLIP scaffolds on HB1^Fluc^ cells incubated in PBS + 0.5 mg/mL collagenase (*n* = 3). (c) Images pre‐ and post‐scaffold implantation in mice. (i) Surgical resection site before implantation of CLIP. (ii) Surgical resection site with CLIP implanted into cavity (white arrow). (iii–iv) Dissected brain tissue 1 month after scaffold implantation. (d) H&E sections from mice implanted with CLIP at 4X (scale bars indicate 500 μm) and 20X (scale bars indicate 100 μm) magnification.

Post‐processing and preparation of 3D printed biomaterials is crucial for mitigating the potential toxicity of unreacted resin components leaching from the printed part. Moreover, toxicity could result from the diffusion of degradation products of the gelatin‐based hydrogel to the seeded cells or surrounding tissues. The in vitro biocompatibility of the scaffolds was evaluated in two toxicity studies. First, the scaffolds were incubated in 1X PBS for 0–56 days to evaluate the toxicity of leachable components (Figure 2a). At specified time points, the PBS was harvested and used to treat HB1^Fluc^ cells for 24 h, after which the BLI of the HB1^Fluc^ was measured. It was found that the viability of the HB1^Fluc^ at all time points were statistically insignificant compared to the control group, HB1^Fluc^ treated with fresh 1X PBS. This indicated the cells were unaffected by any accumulated leachable reactants over time, thus the post‐print wash steps are effective at removing most of the toxic unreacted chemicals inside the CLIP scaffolds prior to use. In a similar experiment, the toxicity of degradation products was assessed (Figure 2b). In this study, CLIP scaffolds were incubated in 1X PBS containing 0.5 mg/mL collagenase enzyme, and the media was replaced twice per week. HB1^Fluc^ viability was measured via BLI after treatment with the degradation media for 24 h. The results of this assay show a statistically significant decrease in viability only for HB1^Fluc^ treated with day 3 scaffold degradation media.

In vivo biocompatibility testing was also completed to evaluate the response to scaffold implantation using our model of mock GBM resection. Athymic nude mice underwent several surgeries, first to expose brain tissue beneath the skull, and next to generate a mock resection cavity by removing a portion of healthy brain tissue, shown in Figure 2c (i–ii). At the time of resection, acellular CLIP scaffolds were implanted into the cavity. The mice were monitored for 1 month with the implants, after which the mice underwent cardiac perfusion to fix and dissect the brain tissue. As shown in Figure 2c (iii–iv), the dissected brain contained the CLIP scaffold at the implantation site. Moreover, there were no obvious signs of inflammation or foreign‐body response in the tissue surrounding the CLIP scaffold.

To evaluate the response at a cellular level, H&E staining was performed on brain sections obtained from mice that underwent scaffold implantation or sham resection surgery (Figure 2d). Notably, mice that received scaffold implants showed substantial macrophage and lymphocyte infiltration around the border of the resection cavity, while only few macrophages could be found remaining in the cavities of sham mice. Importantly, there was no neuronal cell death observed in either condition.

### 3.3 Seeding efficiency and viability of HB1^Fluc^ on CLIP scaffolds

**FIGURE 3:**
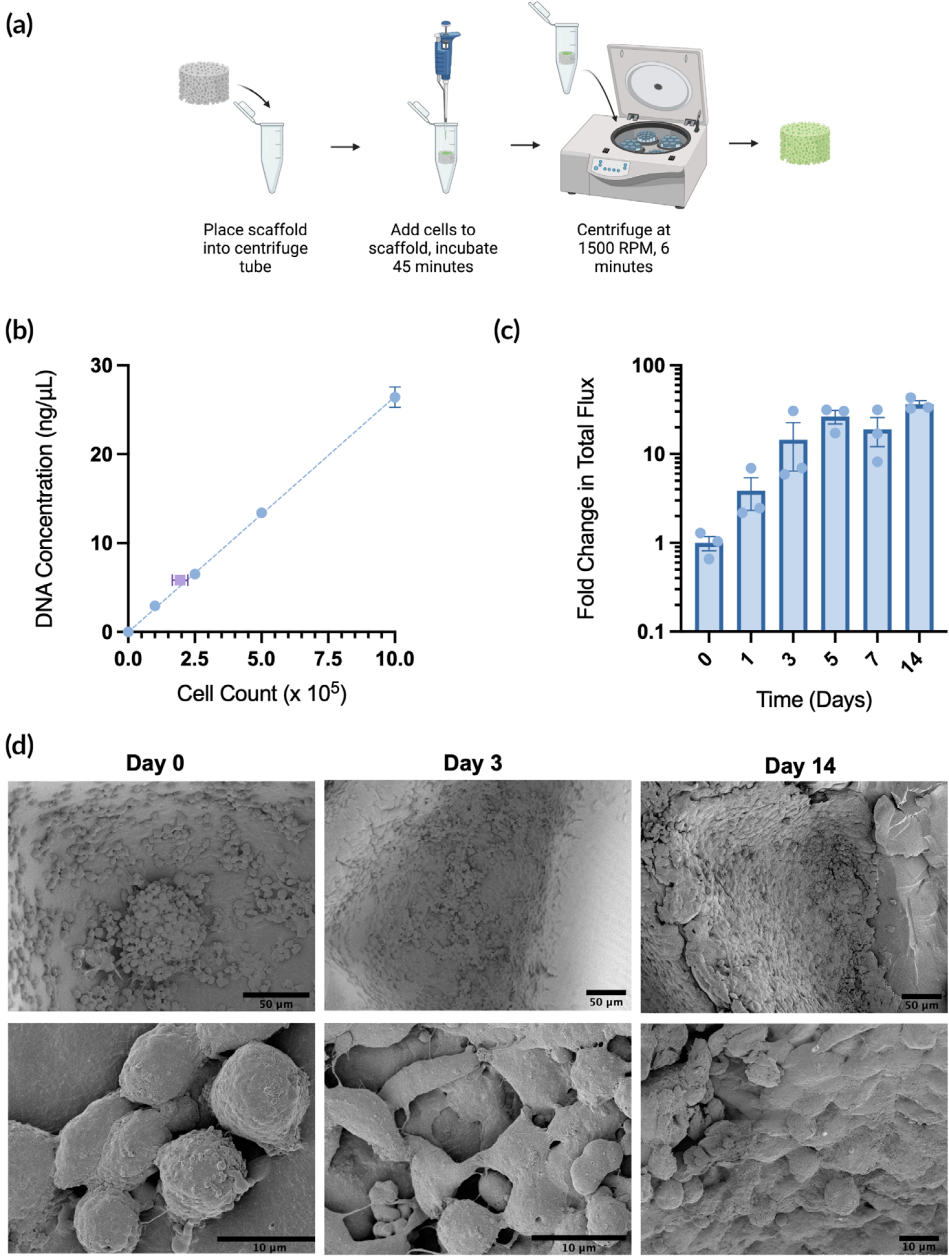
Seeding efficiency and viability of HB1^Fluc^ cells on continuous liquid interface production (CLIP) scaffolds. (a) Schematic of the seeding process. (b) Seeding efficiency of HB1^Fluc^ cells on CLIP scaffolds. Standard curve (*n* = 3) shown in blue, scaffolds (*n* = 3) shown in purple. (c) HB1^Fluc^ viability on CLIP scaffolds (*n* = 3). (d) SEM images of HB1^Fluc^ cells cultured on CLIP scaffolds.

In previous studies published by our group, a dose of 1 × 10^6^ NSCs delivered in saline was sufficient to provide tumor suppression in our mouse model of GBM resection.^15^, ^16^ Based on this dose, our goal was to load the CLIP scaffolds with at least 1 × 10^6^ HB1^Fluc^ at the time of implant. To do so, we adapted a previously published centrifugal seeding strategy to obtain cell‐laden scaffolds (Figure 3a).^34^ First, sterile scaffolds were placed into 2 mL centrifuge tubes, and a 3 μL droplet containing 1 × 10^6^ HB1^Fluc^ suspended in 1X PBS was dispensed onto the top surface of the hydrogel via a micropipette. Scaffolds were incubated with the cell suspension for 45 min at 37°C and 5% CO_2_ to allow the cells to settle onto the surface of the hydrogel. Finally, the tubes containing scaffolds were centrifuged at 1500 RPM for 6 min to pellet the cells into the pockets of the hydrogel. After centrifugation, the scaffolds were added to 6‐well plates containing standard culture media.

Seeding efficiency was evaluated using a DNA extraction assay, in which the DNA concentration obtained from seeded CLIP scaffolds was compared against a standard curve of cell counts and their corresponding DNA concentrations (Figure 3b). It was found that upon initial loading of 1 × 10^6^ HB1^Fluc^, 1.95 ± 0.3 × 10^5^ HB1^Fluc^ were recovered from seeded scaffolds, resulting in a seeding efficiency of 19 ± 3%. Because the cell density on the CLIP scaffolds immediately after seeding did not reach our loading dose goal, we then cultured the scaffolds in standard culture media for 2 weeks. We hypothesized that with additional culture time after centrifugal seeding, the cell density on the scaffolds may increase to the desired loading dose. Moreover, we also hypothesized this culture period may provide the cells time to equilibrate in their new environment, form focal adhesions to the hydrogel, and develop cell–cell interactions.

Cell viability on the scaffolds was measured using the bioluminescence assay shown in Figure 3c. By day 5, HB1^Fluc^ cells on CLIP scaffolds grew to a cell density over 30‐fold higher than that observed immediately after seeding, and this density was maintained until the end of the study. This is reflected by the SEM images shown in Figure 3d, in which notable changes to cell morphology are also observed over time. On day 0, the cells are dispersed sparsely across the scaffold and are mainly spherical in shape. On day 1, the cells are still spherical, but their confluency has greatly increased. By day 3, the cell density is even higher, and the cell morphology has changed to a flattened shape characteristic of adherent cells. This is observed through day 14, on which a layer of cells is observed completely coating the surfaces of the 3D hydrogel.

These results confirm the biocompatibility of the CLIP scaffolds with HB1^Fluc^. The centrifugal seeding method, though it falls short of providing a sufficient cell dose immediately after seeding, provides a cell density that grows rapidly to the desired loading dose upon extra time in culture. The high surface area of the hydrogel permits significant growth of cells over time in culture, allowing for an increase in cell loading despite low initial seeding efficiency. Finally, the surge in observed cell density and the flattened cell morphology observed in SEM images indicates the material provides a satisfactory environment in which the cells can survive for long periods of time. Based on the stabilization of the cell density on the scaffolds by day 5, HB1^Fluc^ were seeded and cultured for 5 days prior to use in all future studies.

### 3.4 Migration of HB1^Fluc^ to GBM from CLIP scaffolds

**FIGURE 4:**
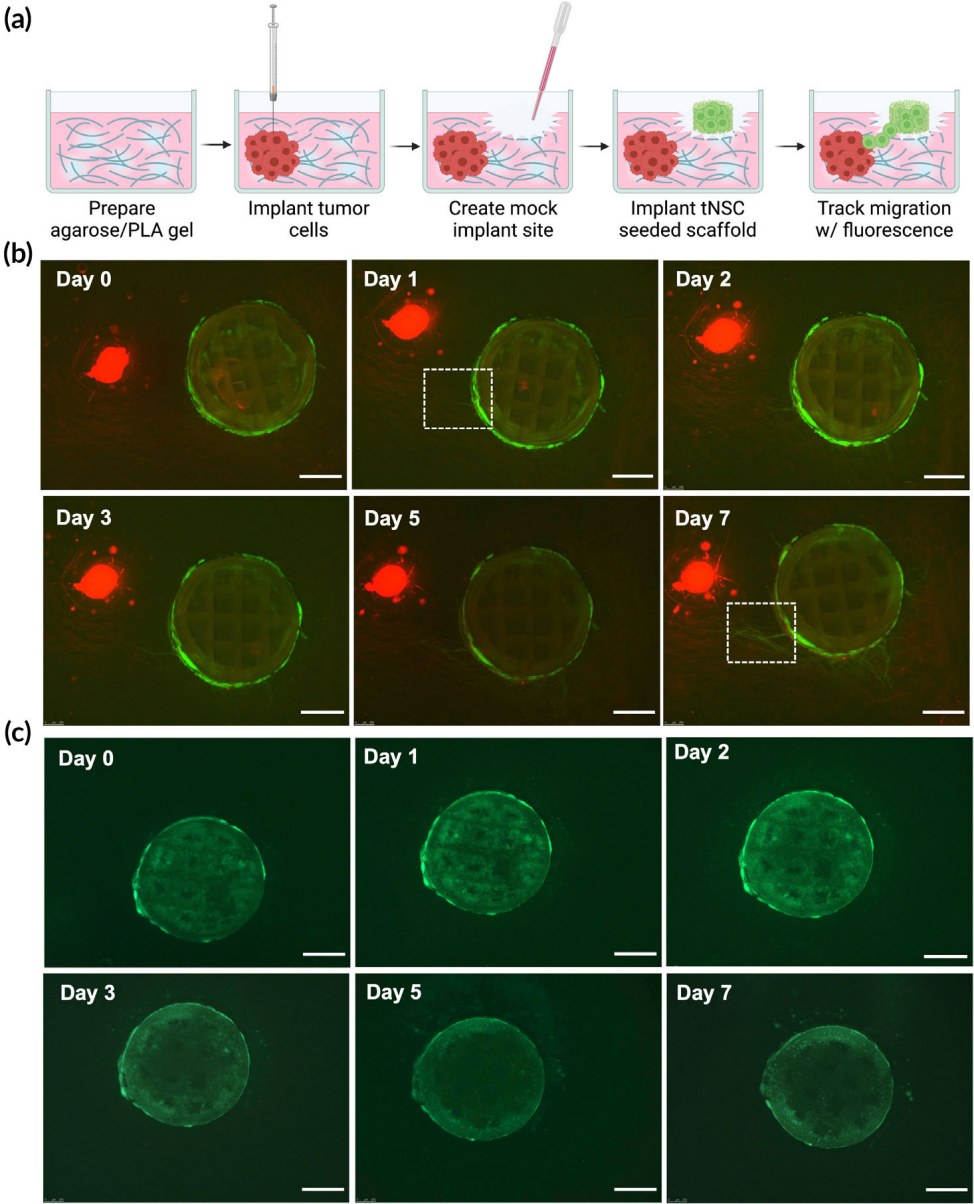
*In vitro* migration assay using a 3D agar culture system. (a) Schematic of 3D agarose gel used to model migration in vitro. (b) Fluorescent images depicting HB1^Fluc^ cells (green) migrating towards LN229^Fluc^ cells (red). (c) Fluorescent images depicting HB1^Fluc^ cells (green) on continuous liquid interface production (CLIP) scaffolds in the absence of adjacent tumor. Scale bars indicate 1 mm.

We hypothesized that in utilizing externally seeded CLIP scaffolds as a cell delivery system, cell migration towards tumor targets in surrounding tissue would not be impeded as it would be if the cells were encapsulated within a biomaterial matrix. It is postulated that cytokines and growth factors produced by the GBM tumor microenvironment, including stromal cell‐derived factor 1 (SDF‐1), monocyte chemotactic protein 1 (MCP1), insulin‐like growth factor 1 (IGF1), and vascular endothelial growth factor (VEGF), act as chemoattractants that induce NSC tumor‐homing.^35^, ^36^, ^37^ As the migratory properties of the HB1.F3.CD cells have been extensively characterized in other publications, the main concern of our study was to confirm that cells can escape from the scaffold and move into the surrounding tissue regions. To test this, we used a 3D agarose gel system to evaluate cell migration from the scaffold in vitro (Figure 4a). Low serum standard culture media (2% FBS) was added to the agarose gel to mitigate the impact of cell proliferation during the assay. As shown in the fluorescence images in Figure 3b, CLIP scaffolds seeded with GFP‐expressing HB1^Fluc^ were cultured for 5 days then implanted into the gel adjacent to a cluster of mCherry‐expressing LN229^Fluc^ tumor cells. Immediately after scaffold implantation, the HB1^Fluc^ GFP signal is entirely contained within the borders of the CLIP scaffold. However, after just 24 h, some HB1^Fluc^ can be observed trailing onto a PLA fiber heading in the direction of the LN229^Fluc^ cells (Figure 4b, Day 1, white box). After 1 week, the HB1^Fluc^ can be observed at a high density on the PLA fibers between the tumor cluster and CLIP scaffold (Figure 4b, Day 7, white box). This trend of HB1^Fluc^ moving down the PLA fibers towards the tumor served as evidence that CLIP scaffolds do not block migration of seeded and cultured HB1^Fluc^. Meanwhile, control images (Figure 4c) demonstrate that while some cells appear to be present outside of the scaffold borders by Day 7, these cells do not appear to be migrating. Rather, it was hypothesized that during the implantation process, some cells may have been physically detached from the scaffold, and then proliferated slowly throughout the duration of the study.

### 3.5 *In vivo* persistence of HB1^Fluc^ on CLIP scaffolds

**FIGURE 5:**
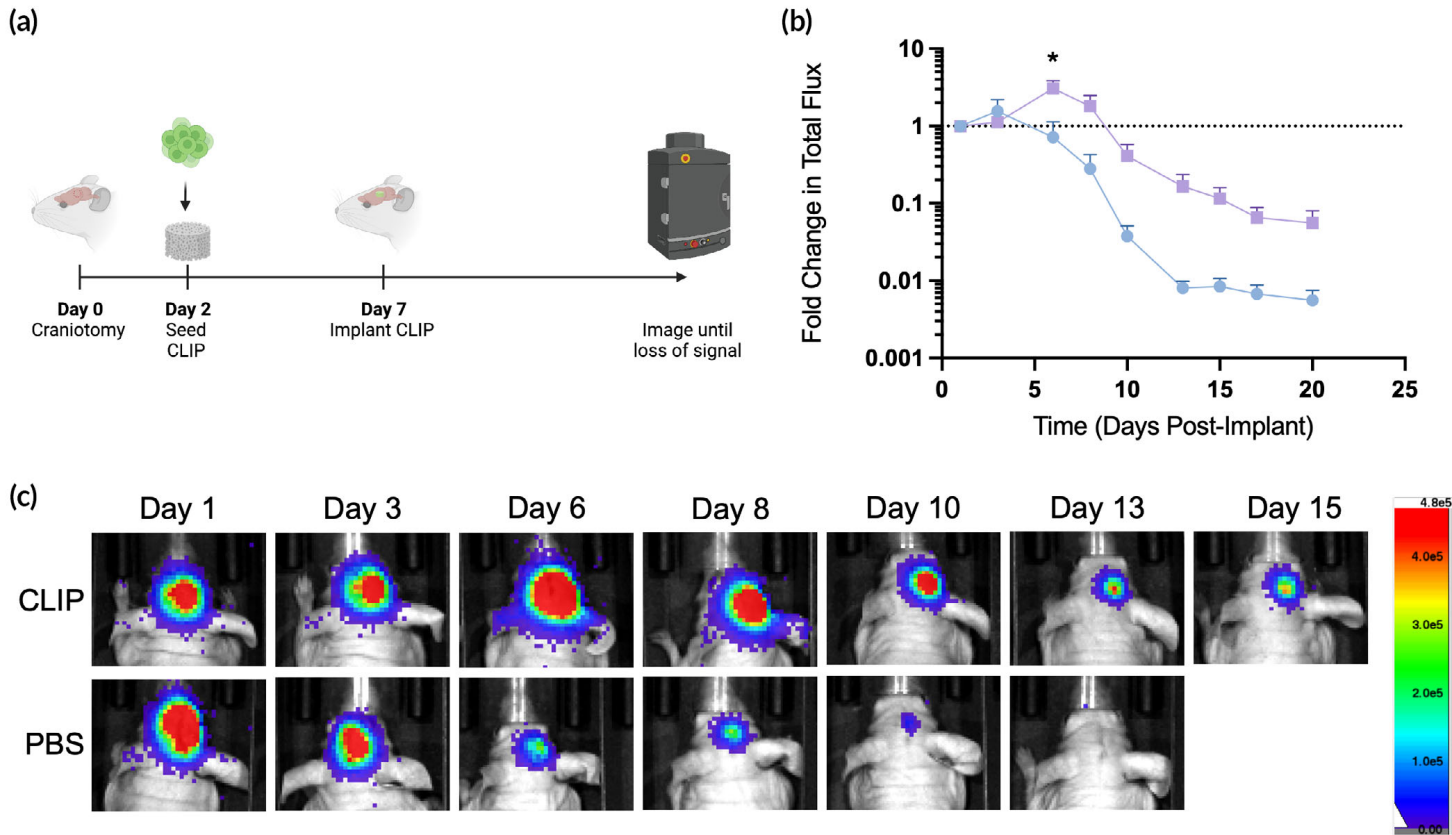
*In vivo* persistence of HB1^Fluc^ cells on continuous liquid interface production (CLIP) scaffolds. (a) Schematic of surgical timeline for the in vivo persistence study. (b) Fold change in HB1 cell BLI signal over time for PBS (blue, *n* = 4) and CLIP scaffolds (purple, *n* = 5). (c) Representative BLI images of mice from each treatment group over the course of the persistence study.

Given that in vitro data suggests that the CLIP scaffolds are biocompatible, support growth of cells to high cell density, and do not impede cell migration, we next used our model of mock GBM resection to evaluate if the scaffolds can improve the survival of therapeutic cells in the surgical site. 1 × 10^6^ HB1^Fluc^ cells were seeded on CLIP scaffolds as previously described, then cultured for 5 days prior to implantation in athymic nude mice (Figure 5a). After implantation, HB1^Fluc^ persistence was monitored using BLI imaging. As shown in Figure 5b,c, HB1^Fluc^ on CLIP exhibited a period of growth lasting 6 days before the BLI signal began to diffuse slowly over time. Alternatively, HB1^Fluc^ implanted in 1X PBS exhibited an immediate and more rapid decline in BLI signal which continued until loss of signal was observed at 2‐weeks post implantation. For the PBS group, over 70% of the BLI signal was lost in 6 days, whereas of BLI signal in the CLIP group had increased by 20%, a statistically significant improvement in persistence.

### 3.6 Efficacy of NHF1^TRAIL^ ‐laden CLIP scaffolds against GBM resection mouse model

**FIGURE 6:**
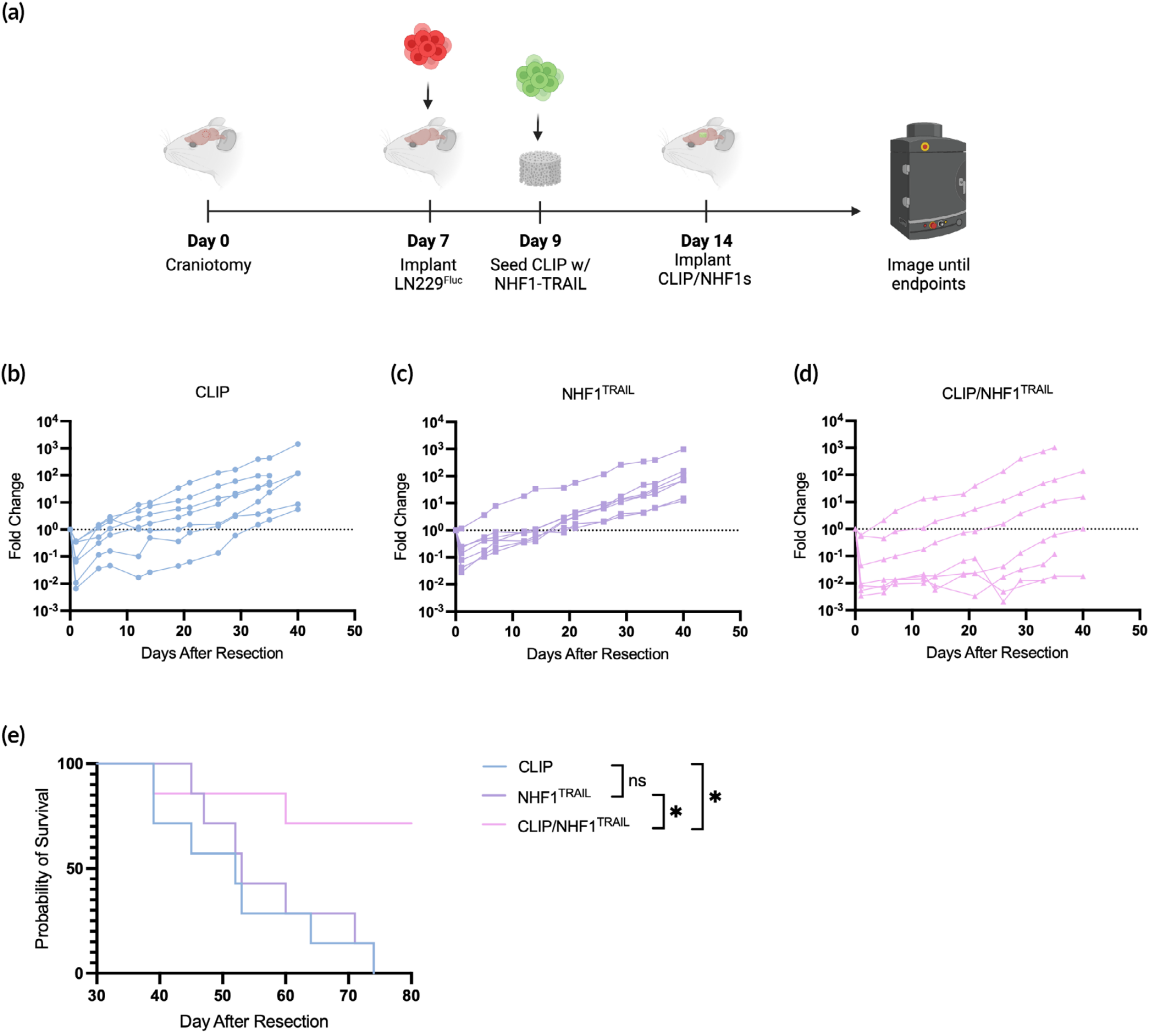
*In vivo* efficacy of NHF1^TRAIL^ cells against LN229^Fluc^ tumors when directly injected into the tumor resection cavity or implanted after external seeding on continuous liquid interface production (CLIP) scaffolds. (a) Schematic of surgical timeline for the in vivo efficacy study. (b) Individual fold change in LN229^Fluc^ total flux values for the CLIP only control group (*n* = 7). (c) Individual fold change in LN229^Fluc^ total flux values for the NHF1^TRAIL^direct injection control group (*n* = 7). (d) Individual fold change in LN229^Fluc^ total flux values for the CLIP/NHF1^TRAIL^ group (*n* = 7). (e) Kaplan–Meier survival curve of mice with LN229^Fluc^ tumors.

Next, we wanted to determine if externally seeded therapeutic cells on CLIP scaffolds can more effectively suppress tumor growth and improve survival outcomes in a murine model of GBM resection. For this model, we used NHF1 cells engineered to constitutively secrete TRAIL, a protein that binds to death receptors on GBM cells to induce apoptosis,^38^ against LN229, an established GBM tumor line that exhibits diffuse behavior *in vivo* (Figure 6a).^39^ In this case, the therapeutic cells are non‐tumor‐homing, but act as a drug pump, in which enhanced cell survival supported by the scaffold allows for a more potent dose of drug to diffuse to surrounding tissue and control tumor recurrence. One week after implanting tumor cells into the brains of athymic nude mice, tumors were resected, and treatments were administered. The treatment groups included acellular CLIP scaffolds, NHF1^TRAIL^ cells injected directly into the resection cavity, and NHF1^TRAIL^ cells seeded on CLIP scaffolds in the same manner as HB1^Fluc^, as described above. BLI imaging was used to track tumor growth over time until significant numbers of began to reach humane endpoints. The fold change in tumor burden, as measured by total flux, of the acellular CLIP group (Figure 6b) began rapidly increasing roughly 12 days after resection, while the NHF1^TRAIL^ (Figure 6c) and CLIP/NHF1^TRAIL^ (Figure 6d) groups showed lower overall tumor burdens until growth rates increased around 20 and 26 days, respectively. Though the trends in total flux were not significant among the treatment groups, significant differences in the survival curves were observed (Figure 6e). The median survival of the NHF1^TRAIL^ group was 53 days, an insignificant improvement over the acellular CLIP group, with a median survival of 52 days. In contrast, the CLIP/NHF1^TRAIL^ group showed a statistically significant improvement in median survival over the CLIP group, suggesting that the ability for cells to persist longer on CLIP scaffolds may result in a longer period of tumor suppression before recurrence is observed. By day 74, all mice in the CLIP and NHF1^TRAIL^ groups had died, with 71% of the CLIP/NHF1^TRAIL^ group still alive at day 80.

## 4 DISCUSSION

GBM patients are in desperate need of new, more effective therapies. There has been no significant improvement in clinical outcomes in almost 20 years, owed to the lack of successful GBM drug development.^40^ Currently, the extent of tumor resection is the greatest predictor of patient survival,^41^ and there is no established standard of care for GBM recurrence.^42^ Combatting recurrence continues to be a challenging effort in the clinical workflow of GBM, as effective drug targets and drug delivery are continued barriers to successful treatment. Chemotherapies that can cross the blood–brain barrier are often highly toxic and demonstrate poor tolerability.^43^ Due to the inherent heterogeneity of GBM tumors, targeted therapies are rarely successful and can lead to drug‐resistant tumor cell populations, making treatment more difficult.^44^ Finally, the immune‐suppressive microenvironment of GBM renders most immunotherapies ineffective.^44^

Cell therapies for GBM have many advantages over existing and developing GBM treatment strategies, but barriers to success still exist. Most importantly, cell delivery and therapeutic durability must be improved for therapeutic success. With most recurrent GBM tumors presenting within 2 cm of the primary tumor, local delivery of cell therapies in the brain is ideal for minimizing toxicity and maximizing tumor suppression.^45^ However, implantation of cell therapies into the brain is an invasive procedure, thus the ability to provide effective and durable treatment with a single, long‐acting dose would be preferred. Moreover, delivery of cells at the time of tumor resection would minimize the total number of required surgical procedures. However, we have previously observed that the persistence of therapeutic cells, namely therapeutic NSCs, in the surgical cavity is poor, likely due to immune‐mediated clearance and physical cell washout by fluid flow into the cavity.^15^, ^16^ A delivery strategy that enhances cell persistence could improve therapeutic efficacy, with the caveat that cell migration and drug release should not be impeded as a result.

While there are plenty of examples in literature detailing biomaterials used to deliver cells into various tissues in the body, much of these systems are utilized for tissue engineering and regenerative medicine purposes.^46^ In these examples, cell retention at the implant site is desired, with the material acting as a protective barrier and guide for cell growth and differentiation. For cell therapy against GBM, cell and drug escape from the delivery system to invasive GBM lesions throughout the brain is vital for successful and durable tumor suppression. However, cells encapsulated in a biomaterial matrix are only able to escape if the material network is degradable or has a large pore size. Previous work indicates that a close relationship exists between degradation rates and tumor suppression post‐resection.^15^ If degradation is too rapid, the cells will be released into the harsh surgical cavity and cleared before they can provide a therapeutic effect. If degradation is too slow, the tumor may exhibit uncontrolled growth until the cells are released, at which point the tumor may be too large for the cells to overcome.

We thus hypothesized that seeding the cells on the external surfaces of a biomaterial system would combine the advantages of protection from clearance and unrestricted migration. Using CLIP, we designed a 3D scaffold with these goals in mind. The 3D lattice architecture allows for cellular ingrowth into the interior of the lattice, where cells will be least likely to encounter immune cells and physical disruption from fluid flow in the cavity. The scaffold also acts as a 3D cell culture environment, where the cells can be “primed” in their new niche prior to implantation in vivo. Literature has shown that priming cells in the material prior to administration can improve in vivo persistence by allowing the cells to produce protective extracellular matrix (ECM) and to form strong cell–cell and cell‐ECM interactions.^19^ Combined, we hypothesized these design features would promote better persistence than therapeutic cells administered in saline alone.

We first showed that the resin described could be used to print the lattice structure, with high resolution observed in the XY plane. However, resolution in the Z‐direction was noticeably poor, leading to overcuring and filling of the intended void spaces of the lattice. Though this pocket‐type feature became advantageous in the subsequent cell seeding process, improving printability of the resin is vital for expanding the portfolio of scaffold designs for this application. To address overcure in the Z‐axis, a water‐soluble UV absorber could be added to the resin, given that this does not impact cell viability on the material post‐printing.^47^

Given that the material is a composite containing degradable GelMA and non‐degradable PEGDA, it is expected that the material would exhibit slow and minimal degradation in vitro. It is possible that the *in vitro* degradation would have plateaued at a certain point beyond the 28‐day experimental timeline, as the PEGDA network cannot be degraded enzymatically. These results agree with the observations from the in vivo biocompatibility study, in which the structural fidelity of the scaffold is largely maintained 1 month after implantation. The swelling characteristics of the material are also expected, given the high water content of the printing resin. Luckily, swelling of the material to equilibrium immediately after printing is minimal, indicating the size and shape of the scaffold will not be dramatically impacted nor be larger than the resection cavity dimensions for in vivo implantation. Additionally, swelling post‐printing did not appear to impact the attachment or proliferation of seeded cells. The mechanical properties of the material agree with the high percentage of low molecular weight PEGDA in the resin, which creates a very stiff and brittle material exhibiting a high Young's modulus. This value is in stark contrast to the Young's modulus of human brain tissue, which is in the range of 0.1–16 kPa, and could potentially be a driver of chronic inflammation at the implant site.^48^ Despite the non‐ideal mechanical properties of the material, this did not majorly impact the handleability of the scaffolds during experiments, nor did the stiffness impact the ability for cells to attach and proliferate on the material.

The toxicity of printed, washed, and sterilized scaffolds was shown to be minimal. *In vitro* studies showed that leached and degraded byproducts from the scaffolds were generally nontoxic, apart from degradation products on day 3. However, toxicity from degradation products were insignificant at all other time points. Moreover, HB1^Fluc^ viability on the scaffolds was maintained for 1 week *in vivo*, and H&E revealed no cytotoxic effects on brain tissue exposed to the material after 28 days. Although considerable macrophage infiltration was observed in mice implanted with CLIP scaffolds, this was an expected immunological response to the biomaterial. Future work will investigate the role of these macrophages in the surgical cavity, specifically in an immune‐competent mouse model, as there is some evidence that gelatin may modulate the polarization of macrophages, via integrin‐mediated interactions or substrate stiffness.^49^, ^50^, ^51^, ^52^ While previously published literature suggests our stiff material may cause macrophage polarization to a pro‐inflammatory phenotype, a more complete understanding of the foreign body response to the scaffold in the brain is needed.

Though immediate seeding efficiency on the scaffolds was relatively low at 19%, viability assays demonstrated HB1^Fluc^ proliferation to high density within 1 week. We hypothesized that by pre‐culturing the cells on the scaffolds prior to implantation, the effective dose of cells delivered in vivo could be increased. As previously mentioned, this strategy could also serve as a priming period in which the HB1^Fluc^ can form stronger attachments to the material and with other cells. Thus, we theorized that a 5‐day culture period between seeding and *in vivo* implantation would facilitate improved *in vivo* persistence and efficacy. The results of the persistence study agree with this hypothesis, with HB1^Fluc^ on CLIP showing significantly higher persistence at day 6 compared to HB1^Fluc^ delivered in saline. In addition, migration studies confirmed that while the cells are perhaps more firmly adhered to the scaffold when implanted after 5 days of culture, this priming period does not interfere with their ability to exit the scaffold and migrate in response to a nearby tumor mass.

Using a model of GBM resection in mice, we demonstrated the ability for CLIP scaffolds to improve the efficacy of a cellular drug pump. We hypothesized that the improvement in tumor suppression and extended period of survival shown in the CLIP/NHF1^TRAIL^ was due to the ability of CLIP to support cell survival and proliferation *in vivo*, which extends the therapeutic window in which TRAIL is being actively produced in the brain. Moreover, secreted TRAIL can easily diffuse through nearby tissue without encountering additional diffusion barriers, since the cells are attached to the external surfaces of the scaffold rather than encapsulated within the material. In addition, previous studies have demonstrated that LN229 may exhibit some level of TRAIL resistance.^53^ This may support the survival results of the efficacy study, in which the difference in survival between the CLIP and NHF1^TRAIL^ groups was insignificant, but the survival of the CLIP/NHF1^TRAIL^ group was significantly longer compared to both other groups. A potential explanation for this result is that the survival of the NHF1^TRAIL^ cells in the brain without CLIP is too short to provide a potent enough dose to kill the tumor cells, leading to eventual recurrence as the tumor develops TRAIL‐resistance mechanisms. In contrast, the surviving dose of NHF1^TRAIL^ cells on CLIP scaffolds after implantation is and remains large enough to kill most of the tumor before resistance can develop. While this data are promising, future testing in additional GBM models, including syngeneic models using immunocompetent mice, models that may exhibit varying levels of sensitivity to TRAIL, and models that replicate the heterogeneous nature of GBM, should be explored to gain a more complete understanding of the influence of CLIP scaffolds on the efficacy of cell therapies against GBM.

## 5 CONCLUSIONS AND PERSPECTIVES

In this study, we demonstrate the development, characterization, and use of a biocompatible scaffold created with CLIP for the purpose of enhancing cell therapies intended for GBM treatment. We demonstrate that NSCs survive, proliferate, and migrate from CLIP scaffolds in vitro, and that therapeutic fibroblasts are more effective *in vivo* when seeded and delivered to the brain on CLIP versus when they are directly implanted in the GBM resection cavity. These promising results prove the potential for this novel CLIP scaffold to act as a platform for the delivery of cell therapies to treat cancer, supporting the need for additional investigation and optimization of the biomaterial formulations and further characterization of the foreign body response in an immune‐competent mouse model. In addition, further optimization of resin formulation and the CLIP printing parameters could enable higher resolution structures to be generated, expanding the potential portfolio of scaffold designs. Future testing may include isolating the effect of various design criteria, including scaffold shape, surface area, and microarchitecture, on modulus, cell seeding, cell migration, drug release, and overall therapeutic efficacy in multiple GBM models.

## AUTHOR CONTRIBUTIONS

**Lauren Kass:** Conceptualization; data curation; formal analysis; investigation; methodology; writing – original draft; writing – review and editing. **Morrent Thang:** Data curation; formal analysis; investigation; methodology; writing – review and editing. **Yu Zhang:** Methodology; writing – review and editing. **Cathleen DeVane:** Investigation. **Julia Logan:** Investigation; methodology. **Addis Tessema:** Conceptualization; investigation; methodology. **Jillian Perry:** Conceptualization; funding acquisition; methodology; supervision; writing – review and editing. **Shawn Hingtgen:** Conceptualization; funding acquisition; methodology; supervision; writing – review and editing.

## CONFLICT OF INTEREST STATEMENT

The authors declare no conflicts of interest.

### PEER REVIEW

The peer review history for this article is available at https://www.webofscience.com/api/gateway/wos/peer‐review/10.1002/btm2.10676.

## Data Availability

All data needed to evaluate the conclusions made in this paper are presented in the paper. Additional data may be requested from the authors.
